# Encoder-Decoder Optimization for Brain-Computer Interfaces

**DOI:** 10.1371/journal.pcbi.1004288

**Published:** 2015-06-01

**Authors:** Josh Merel, Donald M. Pianto, John P. Cunningham, Liam Paninski

**Affiliations:** 1 Neurobiology and Behavior Program, Columbia University, New York, New York, United States of America; 2 Statistics Department, Columbia University, New York, New York, United States of America; 3 Statistics Department, University of Brasília, Brasília, Distrito Federal, Brazil; University of Connecticut, UNITED STATES

## Abstract

Neuroprosthetic brain-computer interfaces are systems that decode neural activity into useful control signals for effectors, such as a cursor on a computer screen. It has long been recognized that both the user and decoding system can adapt to increase the accuracy of the end effector. Co-adaptation is the process whereby a user learns to control the system in conjunction with the decoder adapting to learn the user's neural patterns. We provide a mathematical framework for co-adaptation and relate co-adaptation to the joint optimization of the user's control scheme ("encoding model") and the decoding algorithm's parameters. When the assumptions of that framework are respected, co-adaptation cannot yield better performance than that obtainable by an optimal initial choice of fixed decoder, coupled with optimal user learning. For a specific case, we provide numerical methods to obtain such an optimized decoder. We demonstrate our approach in a model brain-computer interface system using an online prosthesis simulator, a simple human-in-the-loop pyschophysics setup which provides a non-invasive simulation of the BCI setting. These experiments support two claims: that users can learn encoders matched to fixed, optimal decoders and that, once learned, our approach yields expected performance advantages.

## Author Summary

Brain-computer interfaces are systems which allow a user to control a device in their environment via their neural activity. The system consists of hardware used to acquire signals from the brain of the user, algorithms to decode the signals, and some effector in the world that the user will be able to control, such as a cursor on a computer screen. When the user can see the effector under control, the system is closed-loop, such that the user can learn based on discrepancies between intended and actual kinematic outcomes. During training sessions where the user has specified objectives, the decoding algorithm can be updated as well based on discrepancies between what the user is supposed to be doing and what was decoded. When both the user and the decoding algorithm are simultaneously co-adapting, performance can improve. We propose a mathematical framework which contextualizes co-adaptation as a joint optimization of the user’s control scheme and the decoding algorithm, and we relate co-adaptation to optimal, fixed (non-adaptive) choices of decoder. We use simulation and human psychophysics experiments intended to model the BCI setting to demonstrate the utility of this approach.

## Introduction

Brain-computer interfaces (BCI) allow for a user to control effectors, such as a cursor on a computer screen, by the modulation of neural activity. For this approach to be practical for clinical settings, performance optimization through user learning and decoder tuning is a critical issue (see [1] for review). While it is clear that closed-loop control (i.e. control with user feedback) differs from open-loop, off-line decoding [2], there is much uncertainty as to the benefit of co-adaptation, namely using adaptive decoding algorithms in a setting where the user is also learning. There is optimism that co-adaptation should yield performance better than the simpler scheme of presenting a fixed decoder in closed-loop and user learning, but a rigorous framework for clarifying these issues has been lacking. Here we offer such a theoretical framework. According to this theory, for arbitrary, fixed decoders, performance after the user has learned should differ, but for a correct choice of fixed decoder, only the user needs to learn in order to obtain optimal performance. While this result may initially seem surprising, it becomes apparent when the problem is cast as a joint optimization over encoders and decoders. We begin by reviewing co-adaptation and then motivate our approach.

### Co-adaptation

When the BCI system is closed-loop such that both the user can learn and the decoding algorithm can adapt, we have a setting which permits co-adaptation [3, 4]. Changes in system performance may be driven by distinct types of adaptation: (1) All of the adaptation may occur due to the decoder and the user might not learn on relevant timescales [5]. (2) The user may learn in response to a fixed decoder [6, 7]. (3) Both the user and the decoder might change, but the end result might perform no better than if only either the decoder or the user had learned. (4) In the most fortunate case, co-adaptation might be able to permit some synergistic result where both the user and the decoder learn in a collaborative fashion which yields high performance. Results in the literature hint suggestively at possibility (4), yet it is difficult to distinguish this from possibility (3) because even if both decoder and user adapt, it may not be clear which drove the performance improvements [4]. Here we investigate this open question.

While previous work has examined how decoder parameters should adapt in closed-loop settings in order to improve over static decoders [5, 8, 9], there has not been an emphasis on doing so while explicitly considering how the user adapts. We define the decoder as the external system which decodes neural activity into end effector (BCI) control signals. The encoder then is the user’s internal encoding of intention into neural activity. As a clarifying example, neurons with simple tuning curves imply a linear encoding model in some cases, and a user’s adaptation of the encoder could correspond directly to tuning curve shifts [4, 6, 7, 10]. For a given, fixed decoder, different encoding models will perform differently and better encoders will be those that, in some sense, match the decoder well. We will show that it is possible to compute the “best” encoding model for a given decoder—that is, some encoding model exists that minimizes the mean squared error (MSE) performance for a given decoder subject to signal-to-noise ratio (SNR) constraints. Similarly, for a given encoding model, there exists a best decoder in the MSE sense [11]. When a given decoder is presented to a user, optimal performance will be obtained if the user adapts such that encoding is optimally matched to the decoder. With this knowledge, one might imagine it useful to attempt to shape the user’s neural tuning properties [12] or otherwise attempt to guide the user to a certain encoding scheme which has been determined will optimize performance.

### Motivation for our approach

Conceptually motivated by co-adaptation and BCI-user learning, we propose a framework for jointly optimizing the encoding model and decoder of a BCI under a minimum mean square error (MMSE) objective. The central premise of this work is that closed-loop co-adaptation is a special case of this joint encoder-decoder optimization problem, so an optimal decoder can be computed in advance, effectively circumventing the co-adaptation process. The core of our approach amounts to “pre-computing” the limit of an idealized co-adaptation process in which the user (hypothetically) optimally learns in order to obtain an optimized encoder-decoder pair. Instead of focusing on the temporal dynamics of learning in closed-loop [11], we abstract to the space of encoder-decoder pairs and characterize how well different encoder-decoder pairs perform relative to one another. We pre-compute an optimal decoder which we can present as a fixed decoder to the user; given our modeling assumptions, the performance of this decoder by definition should not be surpassed by one obtained through co-adaptation. We emphasize that closed-loop learning by the user will still be critical to learn to control the fixed decoder, but that the decoder will not also need to be adapted.

In a very general sense, learning by both the user and the decoder is equivalent to an optimization of performance with respect to these components. This perspective is essential, as it allows us to critically investigate co-adaptation as a mathematical optimization procedure, rather than an ill-defined “synergistic” training process. Co-adaptation then amounts to a specific approach to optimizing this objective over time—a coordinate descent approach where the user updates the encoder, and then the BCI system updates the decoder, iteratively. Seen from this perspective, it becomes clear that this objective function over encoder-decoder pairs could instead be descended using some other optimization strategy, and then a fixed, pre-optimized decoder could be presented to the user and learned. Such a setting would obviate co-adaptation. This approach could break down in a few key places: (1) The optimization could be intractable. (2) We may not be able to characterize the user objective. (3) We may not be able to characterize the constraints on user learning.

It is worth emphasizing that these issues are present for co-adaptation as much as any generic optimization approach. Co-adaptation may not do a good job of obtaining optima of the objective function, and without knowing the user’s objective or constraints on user learning, co-adaptive decoder updates may be very difficult to tune or suboptimal.

In the remainder of this work, we specify a MSE objective, and we work through the joint encoder-decoder optimization framework for the case when encoding is linear and the decoder is a steady state Kalman Filter (SSKF). We assume that the SNR of the neurons is constrained and that it is difficult for the user to learn encoding models in which the correlation structure of the neural activity must change too much. We also assume that the user is aware of the MSE objective and is motivated to optimize this objective. Our model form is broadly consistent with other Kalman Filter decoding schemes employed in contemporary BCI. However, in particular settings, the objective function and the constraints on learning of the encoding model could be specialized. We return to the opportunity for specialization in the discussion after presenting our results.

Finally, we validate the pre-computed decoder in computer simulations as well as in an online prosthesis simulator (OPS) [13], a psychophysics platform which can serve as a test-bed for BCI. The OPS demonstrations are fully transparent (e.g. allowing us to specify neural signal and noise characteristics) so they allow us to gain insights into how the approaches we are studying work, and we show that our approach provides decoders which are both plausibly learnable and permit performance improvements on point-to-point reaching tasks.

## Results

### Encoder-Decoder system framework

Allowing for changes to both the decoder and encoder, we here show how to obtain encoder-decoder pairs which theoretically yield better performance than would be obtained either by learning an arbitrary, fixed decoder or adapting the decoder when the user is not learning. Although conceptually applicable generally, we apply our framework to the conventional Kalman filter (KF), which serves as a reasonable choice of decoder for BCI system. KF decoding approaches have been made to have adaptive parameters in various ways [4, 5, 8, 14]. However, no previous work has directly considered the co-adaptation problem as a joint optimization problem over pairs of encoders and decoders. Here we review the KF and then show how to derive optimal encoder-decoder pairs.

#### Review of Kalman Filter for neuroprosthetics

**Fig 1:**
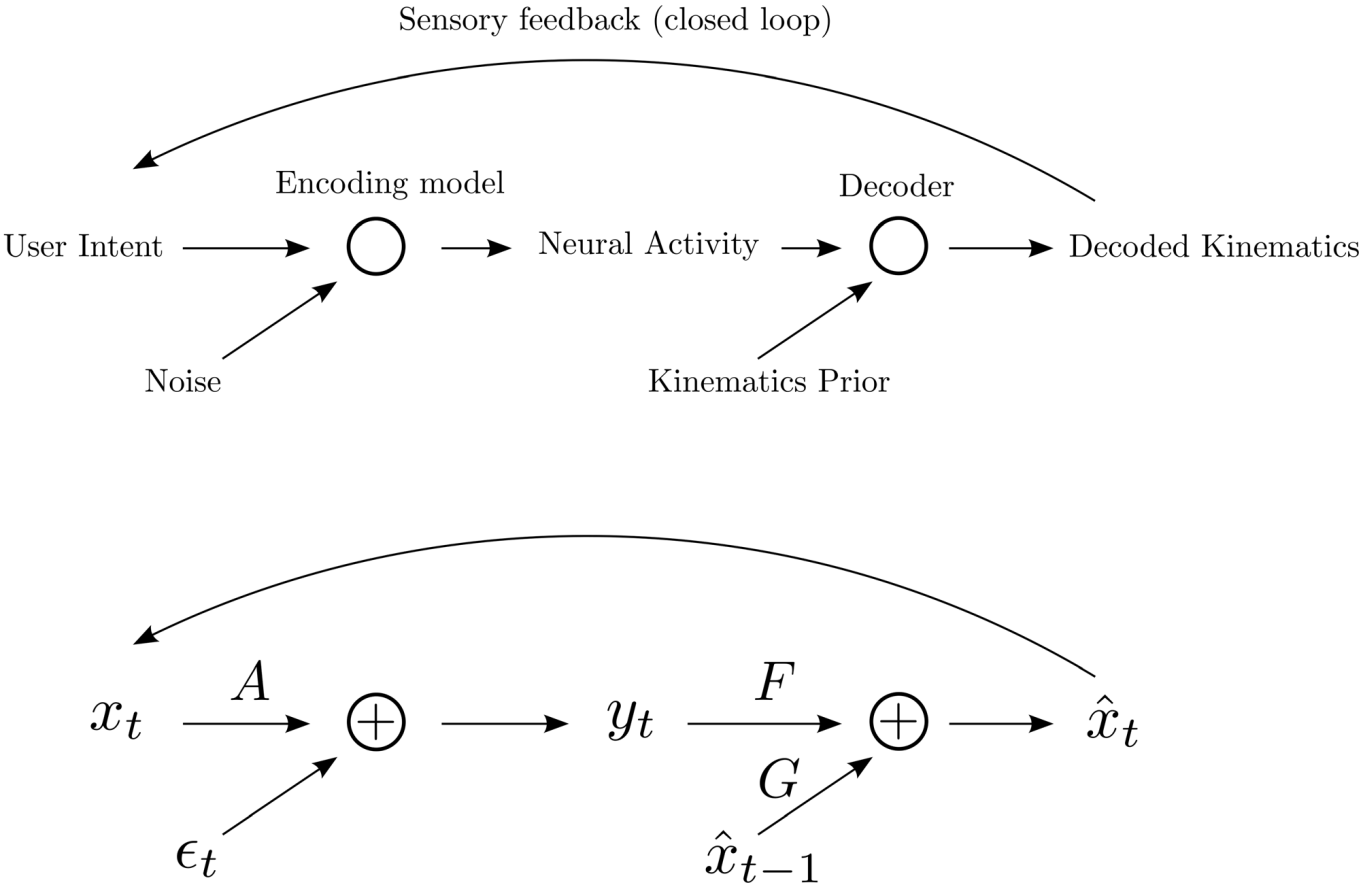
BCI decoding framework. Top figure depicts the general setting where neural activity is taken together with prior information to decode an estimate of the underlying intention. When in closed loop, sensory (visual) feedback is provided which can allow the user to modify intention and also permit changes to the encoding model. Bottom figure depicts the steady state Kalman filter (SSKF), a simple exemplar of the general setting in which *A*, *F*, & *G* are matrices which multiply the vectors *x*_*t*_, *y*_*t*_, or ${\hat{x}}_{t-1}$. Contributions from *Fy*_*t*_ and $G{\hat{x}}_{t-1}$ combine additively.

To build a Bayesian decoder, we will need both an “encoding model” (or encoder), mapping user intention to neural activity, as well as a “prior model”, which relates the variables capturing user intention across time (Fig 1). In the simple case of controlling a cursor on a screen, the encoding model relates user intention about kinematic variables (such as cursor position and velocity) to neural activity. The prior relates these kinematic variables across time, effectively serving to smooth them. The Bayesian decoding algorithm combines the prior and encoding model using Bayes rule to infer the user’s intended kinematics from neural activity [15]. The Kalman filter (KF) is a simple Bayesian decoder and is widely used as a decoder for cursor control neuroprosthetic applications [4, 8, 14–16].

For the KF, the kinematic variables are related by a first order autoregressive process (AR(1)), which serves as a prior model over cursor trajectories.

$$\begin{array}{c} x_{t}=Px_{t-1}+z_{t}\;\text{(with}\;z_{t}∼𝒩\left(0,Q\right)\text{)}. \end{array}$$ (1)

Here *x*_*t*_ is a vector of size *n*, corresponding to the number of dimensions to be controlled (i.e. the kinematic variables corresponding to user intention). *P* is therefore an *n* by *n* transition matrix which characterizes the physical dependencies between variables across timesteps. This prior equation encourages posterior estimates of consecutive time points to be nearby if the noise covariance, *Q*, is small.

We then specify an observation model, which in our case will be the linear encoding of the kinematic variable in the neural activity with additive Gaussian noise.

$$\begin{array}{c} y_{t}=Ax_{t}+\epsilon_{t}\;\text{(with}\;\epsilon_{t}∼𝒩\left(0,C\right)\text{)}. \end{array}$$ (2)

Here *y*_*t*_ is a vector of size *k*, the number of observed neural signals. This observation could be any observation of user intent such as binned spiking activity, electrocorticography, electroencephalography, peripheral electromyography, or other methods. Conceptually the observation matrix *A* (size *k* by *n*) corresponds to the encoding model parameters. For this model, the Kalman filter is known to be the optimal Bayesian decoder and it also minimizes the squared error of the decoded variables with respect to the actual value [17, 18].

The Kalman filter has transients due to initial conditions, but we are interested in the performance of the user and decoder on long timescales, so we will prefer the simplification that the system is in steady state. It has been shown that very little performance is lost in BCI settings using steady state approaches [19]. It is well known that a decoder of the following form corresponds to a steady-state Kalman Filter (SSKF) [20]:

$$\begin{array}{c} {\hat{x}}_{t}=Fy_{t}+G{\hat{x}}_{t-1}. \end{array}$$ (3)

Matrices *F* and *G* correspond to decoding model parameters, and ${\hat{x}}_{t}$ is a vector of size *n*. *F* and *G* depend on the Kalman model parameters *A*, *C*, *P*, and *Q*, and solving for the SSKF parameters is standard [20]. The solution is:

$$\begin{array}{c} F=\Sigma_{SS}A^{T}{\left(A\Sigma_{SS}A^{T}+C\right)}^{-1}. \end{array}$$ (4)

$$\begin{array}{c} G=P-FAP. \end{array}$$ (5)

where Σ_*SS*_ is the fixed point solution to the Riccati equation:

$$\begin{array}{c} \Sigma_{\text{SS}}=P\left(\Sigma_{\text{SS}}-\Sigma_{\text{SS}}A^{T}{\left(A\Sigma_{\text{SS}}A^{T}+C\right)}^{-1}A\Sigma_{\text{SS}}\right)P^{T}+Q. \end{array}$$ (6)

#### Joint optimization of the encoder and decoder

*F* and *G* are parameters of the decoder, so they are free to be selected by the decoding algorithm. *A* corresponds to the user’s encoding model and is not directly selectable by the experimenter. However, assuming the user can learn and is motivated to perform well, presenting a fixed decoder should cause the user to attempt to match the decoder with the corresponding optimal encoding model. Learning here corresponds to exploring the space of mappings between intention and neural activity (captured by *A*), and the user can change *A* by modulating how their intention corresponds to desired control in the kinematic space (i.e. the mapping from “what the user thinks” to “what the end effector does”).

The problem we want to solve then is the joint optimization of the encoder and decoder. In the general Kalman filter decoder setting, we solve for parameters *F* and *G*, but *A* is specified. By solving for *A* also, we are finding the KF solution for an optimal encoder. This joint optimization space is equivalent to that covered by co-adaptive settings in which the KF parameters can adapt and the user can learn—that is, any setting in which the user learns and the decoder is a Kalman filter variant is subsumed by our approach of looking at the whole space of user encoders and KF decoders.

For the problems we consider, we are provided with a source distribution for the kinematic intention variables *x*_*t*_, given by the AR(1) model parameters *P* and *Q* which are assumed known (these can be engineered for the task). We optimize over encoding model parameters *A* and the decoder parameters *F* and *G* with respect to a minimum mean square error (MMSE) objective. The solution depends on the statistics of the neural signal *y*_*t*_, predominantly given by the observation noise (*C*). For the following derivations, we assume the noise covariance (*C*) is known, although it would be estimated in practice. Also, in practice the neural activity would be pre-processed—we assume zero-mean signals.

#### Optimal solution for AR(1) kinematics

In trying to determine the optimal encoder-decoder pair, we consider the objective function to be MMSE, so the objective is (see equations (15)–(18) of S1 Text for more details):

$$\begin{array}{c} ℰ\left(A,F,G\right)=𝔼\left[{\left({\hat{x}}_{t}-x_{t}\right)}^{T}\left({\hat{x}}_{t}-x_{t}\right)\right]=tr\left[𝔼\left[{\hat{x}}_{t}{\hat{x}}_{t}^{T}\right]-2𝔼\left[x_{t}{\hat{x}}_{t}^{T}\right]+𝔼\left[x_{t}x_{t}^{T}\right]\right] \end{array}$$ (7)

$$\begin{array}{c} =tr\left(\left(FA-I\right)\Sigma_{x}{\left(FA-I\right)}^{T}\right)+tr\left(2\left(FA-I\right)𝔼\left[x_{t}{\hat{x}}_{t-1}^{T}\right]G^{T}\right)+tr\left(FCF^{T}\right)+tr\left(G𝔼\left[{\hat{x}}_{t-1}{\hat{x}}_{t-1}^{T}\right]G^{T}\right), \end{array}$$ (8)

where Σ_*x*_ is the marginal covariance for *x*_*t*_ given by the (zero-mean) process distribution in Eq 1:

$$\begin{array}{c} \Sigma_{x}=𝔼\left[x_{t}x_{t}^{T}\right]=P𝔼\left[x_{t-1}x_{t-1}^{T}\right]P^{T}+𝔼\left[z_{t}z_{t}^{T}\right] \end{array}$$ (9)

$$\begin{array}{c} =P\Sigma_{x}P^{T}+Q. \end{array}$$ (10)

This is a Lyapunov equation and the solution is standard, as *P* and *Q* are assumed known. We can solve for Σ_*x*_ (and thereby the marginal distribution *x*_*t*_ ∼ 𝒩(0, Σ_*x*_)) by iterating the matrix recursion to convergence or by vectorizing and solving for the covariance in closed form [21].

The choice of encoding *A* determines the signal strength in the observed neural data *y*_*t*_, which also carries an additive noise component with covariance *C*, as shown in Eq 2. If we simply attempt to optimize encoding and decoding for the MMSE objective in an unconstrained fashion, we will obtain the trivial solution of *A* → ∞. Such a solution corresponds to arbitrarily high firing rates of neurons.

To capture the physiological requirement that neurons cannot be active with arbitrary magnitude and/or arbitrarily low noise, we must penalize the signal power while encouraging it to be as distinct as possible from the noise power. Additionally, when considering the optimal encoder-decoder pair, we want to constrain the optimal encoding model to a set which is feasible for the user to learn. Several studies suggest that BCI users can only adjust neural activity such that it does not violate certain features of the neural activity structure [22–24]. To capture the basic structure of some of these limitations, we can place a simple phenomenological constraint on the encoder which prohibits the covariance of the neural activity from deviating too much from an initial, native setting. Specifically, a standard approach for multivariate signals is to use a trace-of-quotient penalty—this is also used in linear discriminant analysis and other methods that share this motivation [25].

$$\begin{array}{c} {𝒢}_{joint}\left(A\right)=tr\left(𝔼{\left[y_{t}y_{t}^{T}\right]}^{-1}𝔼\left[\left(Ax_{t}\right){\left(Ax_{t}\right)}^{T}\right]\right) \end{array}$$ (11)

$$\begin{array}{c} =tr({\hat{\Sigma }}_{y}^{-1}A\Sigma_{x}A^{T}) \end{array}$$ (12)

where

$$\begin{array}{c} \Sigma_{y}=𝔼\left[y_{t}y_{t}^{T}\right]=\underset{\Sigma_{sig}}{\underset{︸}{A\Sigma_{x}A^{T}}}+C. \end{array}$$ (13)

For our class of models, this joint activity constraint ensures that both signal and noise correlations in neural activity are appropriately respected. If future experiments reveal additional constraints that restrict the set of encoding models learnable by the user, it would be straightforward to model them and incorporate them into an updated penalty. In the simpler case where neural signal constraints are unknown and we only wish to focus on noise constraints, we can replace Σ_*y*_ with *C* yielding 𝒢_*SNR*_(*A*) = *tr*(*C* ^−1^ *A*Σ_*x*_ *A* ^*T*^).

We add the 𝒢_*joint*_(*A*) penalizer to our objective and arrive at the main optimization problem we are trying to solve. The full objective for the minimum MSE encoder-decoder pair subject to this penalty on the encoding scheme is:

$$\begin{array}{c} ℒ{\left(A,F,G\right)}^{*}=ℰ\left(A,F,G\right)+\lambda {𝒢}_{joint}\left(A\right). \end{array}$$ (14)

The *λ* parameter enforces a specific magnitude of the constraint (which can be tuned as a Lagrange multiplier to match the SNR of the actual system). This objective function serves as a key theoretical statement of this paper insofar as it describes a well-defined optimization problem that summarizes optimal co-adaptation.

We optimize the full objective (Eq 14) with respect to *F*, *G*, and *A*. For *A* held fixed, the optimal *F* and *G* follow from standard methods for solving for the optimal SSKF (i.e. linear-quadratic estimator). For *F* and *G* held fixed, we can take explicitly the gradients of this objective with respect to encoding model parameters *A*. We alternate between gradient-based optimization of *A* given *F*, *G* and optimization of *F*, *G* given *A*—this is a coordinate-wise optimization of the full objective to obtain a joint local optimum (see S1 Text for full derivation).

#### Characterization of solutions

Conceptually, we compare the optimal coding solutions of the form described in the previous section with those that follow from using a static, linear decoder instead of a Kalman filter:

$$\begin{array}{c} {\hat{x}}_{t}=Fy_{t}. \end{array}$$ (15)

Such a decoder corresponds to the setting where each timestep of the process *x*_1…*T*_ is sampled independently and identically distributed (i.i.d.) from a Gaussian, rather than from an AR(1) process. Work on “robust coding” has solved the optimal encoder-decoder problem for i.i.d. input without temporal correlations—versions of this problem are explored in the context of neuroscience in [26–28], and an abstract variant of this problems was solved much earlier in the control literature [29]. If we consider only the marginal statistics of our kinematic variables (which are Gaussian), the solution of our problem aligns with the classical robust coding solutions (see S1 Text for details). The solution to the full problem from the preceding section appropriately generalizes this marginal solution. In the limit of *P* → 0 which eliminates the temporal dependencies, the KF-decoder solution turns into this marginal solution (see S1 Text).

For the AR(1) case, we have empirically studied the objective function using both penalties with up to k = 200 neural channels to control n = 3 dimensions. As long as parameters are initialized to the correct order of magnitude (for numerical stability), optimized encoders and decoders obtained from different, random initializations converge to the same value of the objective function. The parameters (*A*, *F*, *G*) obtained are different but the invariant objective function value suggests that most local optima may comprise a set of solutions which obtain the same error. The observation of multiple equivalent optimal solutions is consistent with the various indeterminacies in our problem—that is, ℒ(*A*, *F*, *G*)* does not change if the encoder *A* and the decoder *F* are both operated on in ways which preserve the quantities in the objective function. In this work, we only focus on situations where the kinematic variables correspond to position, so *P* ∝ *I*_*n* × *n*_ and Σ_*x*_ ∝ *I*_*n* × *n*_. In general, if there are other variables such as velocities, then *P* will have a block which is ∝ *I*_*n* × *n*_ and other blocks which relate the variables to position according to physical relationships (see [30] for an explicit presentation of this). Choices of parameters (such as a structured *P* and a full rank signal covariance with the 𝒢_*joint*_(*A*) penalty) can break most of the indeterminacies, in some cases leaving only a sign indeterminacy (i.e. *F* and *A* may both have signs of parameters inverted with no affect on the objective). Overall, it is clear that our algorithm obtains optima for which performance is not dominated by other encoder-decoder pairs.

### Validation of framework

Having provided the framework and described the encoder-decoder optimization methods, we validate our approach in two settings. We first examine the coding schemes arising from simulations and verify that our approach yields interpretable solutions. Following the simulation results, we show the results of psychophysics experiments implementing our approach on an online prosthesis simulator (OPS), a system which tracks overt human body movements and generates synthetic, closed-loop data.

#### Finding good encoder-decoder pairs (simulation)

In this section, we wish to build intuition for the optimized encoder-decoder pairs we obtain. Consider a generic case in which there are *k* “electrodes,” each having independent signal and noise, and we wish to design a decoder having only a single output degree of freedom (DOF), a 1D ${\hat{x}}_{t}$. In this case, the encoder is a *k* × 1 matrix mapping intention *x*_*t*_ to the *k*-dimensional neural observation *y*_*t*_. The decoder is a 1 × *k* matrix mapping from *y*_*t*_ to an estimate ${\hat{x}}_{t}$.

**Fig 2:**
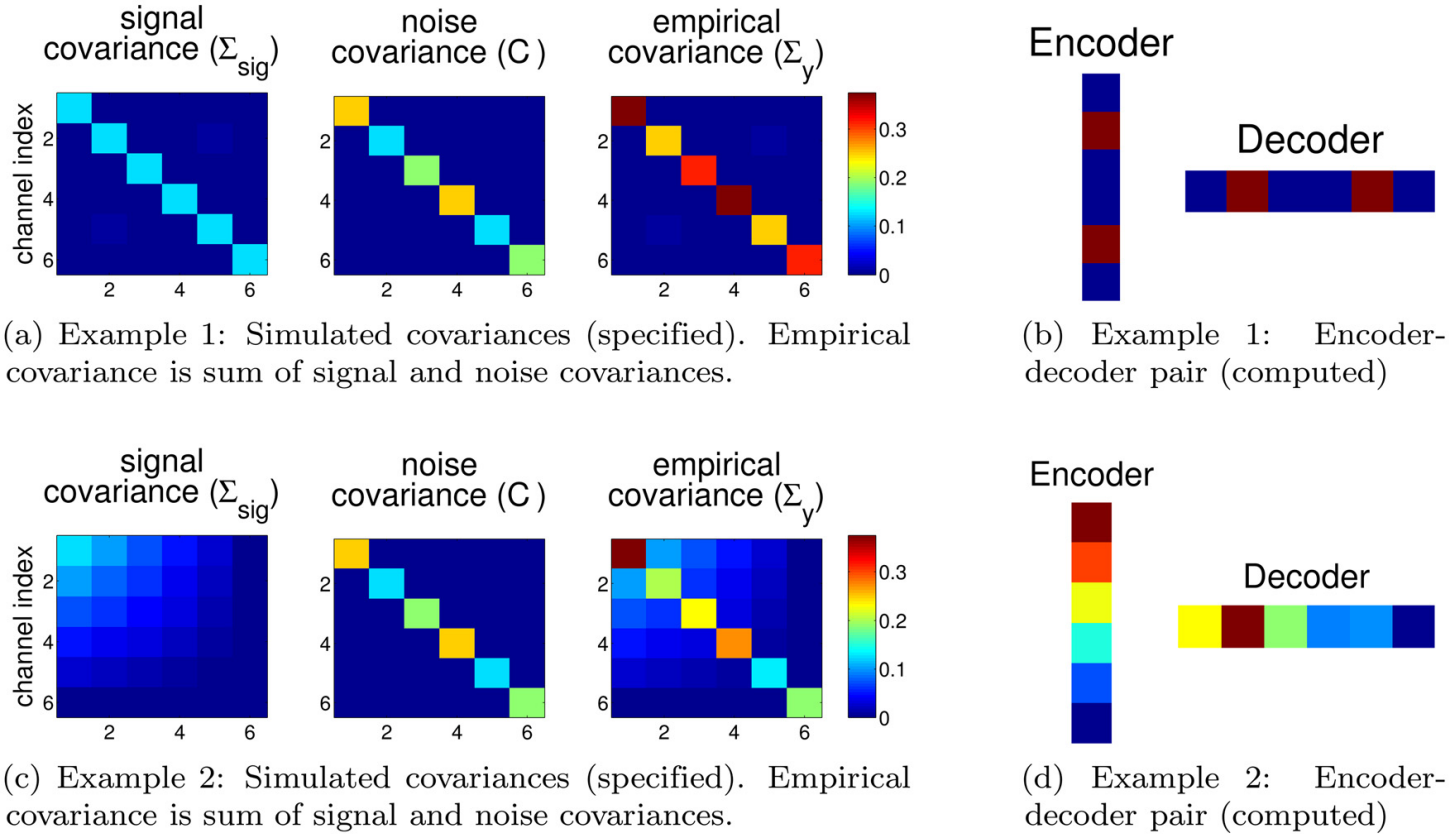
Two simulated examples of signal and noise covariances which give easily interpretable encoder-decoder optima. In (a) & (c) we generate a signal covariance (Σ_*sig*_) and noise covariance (*C*) which sum to the empirical covariance (Σ_*y*_). These covariances are sufficient statistics for the optimization procedure and allow us to determine the optimal encoder-decoder pair for these channels. In (b) & (d) we visualize the corresponding optimal encoder and decoder. For example 1 (a) & (b), the optimized encoder-decoder pair transmits information along dimensions 2 & 5 which have the highest SNR (i.e. signal magnitude is same for all dimensions and noise is lowest for dimensions 2 & 5). For example 2 (c) & (d), the signal is a rank 1 matrix and the encoder parameters preferentially encode higher signal dimensions. The obtained decoder parameters again reflect preferential decoding of dimensions with high signal and low noise.

We can simulate this setting by constructing signal and noise covariance matrices, and we can numerically compute the optimal decoder parameters. The optimization procedure finds encoders which have good signal subject to constraints on the magnitude of the encoded variables (i.e. for fixed SNR level). The encoder corresponds to the *A* matrix, visualized as the encoder in Fig 2. The corresponding decoder parameters *F* should rely on the more strongly encoded dimensions, with more decoder weight on those dimensions which are less noisy. The decoder parameters *G* are also important, but they largely reflect the prior dynamics parameters *P* which in this case are proportional to the identity matrix.

Indeed, in simple example simulations here, we see that signal is coded along dimensions with less noise. In example 1 (top row of Fig 2), signal is present and equally strong for all dimensions, and noise is lowest for dimensions 2 and 5. We optimized the encoder and decoder for the specified covariances, and we see that, consistent with intuitions, optimal encoding and decoding relies primarily on the two, high-SNR dimensions. An optimal decoder may ignore noisy channels in favor of not mixing less reliable channels with the more reliable channels if this is best for the objective function. In example 2 (bottom row of Fig 2), signal covariance reflects decreasing signal on higher numbered dimensions. The noise is the same as in example 1. The optimized encoder encodes the high signal dimensions preferentially and the decoder reads out the dimensions which both have high signal and low noise (e.g. dimension 2 is decoded more strongly than dimension 1 since it is considerably less noisy). In 1D control, the only indeterminacy is of the sign of both the encoder and decoder. Both of these examples validate that our optimization yields encoder-decoder pairs which conform to our intuition of what constitutes a good encoding scheme. If it is believed that the representations available in a specific setting should be subject to additional constraints, these should be included in the objective.

The key point is that the encoder and decoder are matched. Recall that in the prosthetic setting, the user’s initial encoding will be arbitrary, but users who change their encoding (i.e. adapt their control scheme) to match the optimized encoder, will potentially be able to obtain optimal performance when using the corresponding decoder. The feature of our framework, that neurons not relevant for the decoder will have decreased encoding weight, has experimental corroboration in BCI settings from work showing that neurons not used by a decoder have decreased modulation [31, 32].

#### Online prosthesis simulation for 1D control

**Fig 3:**
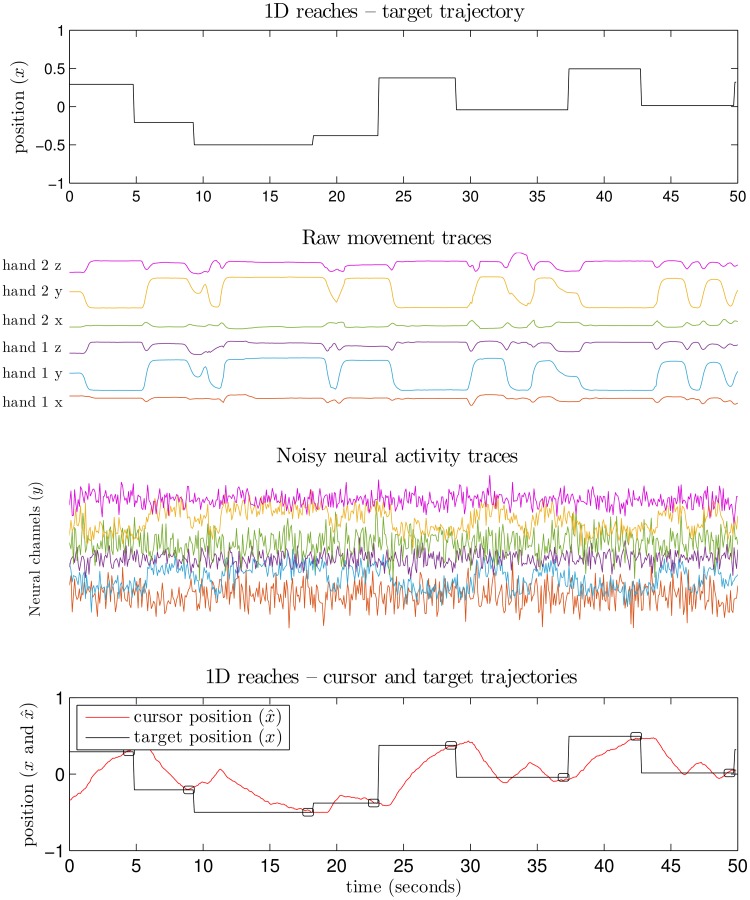
Pipeline for OPS experiments with a single-trial trace for the “pinball” (point-to-point reaching) task (subject 2, SNR-case 3, using the pre-computed decoder). The top-most plot depicts target position observed by user, in response to which the user makes overt movements reflecting their intention. The second plot depicts these overt user movements captured by the motion-capture sensor and pose tracking software. The third plot depicts simulated neural activity which was synthesized by reweighting, mixing and adding noise to the raw movement data. The bottom-most plot depicts decoded cursor positions (1D) during the task. Target remains fixed until acquired by the cursor, noted by black markings around target transition times. Trajectories are smooth and under/overshoots are common, visual feedback being crucial to performance.

In order to thoroughly examine our approach when we know how neural activity is generated, we tested our framework with human subjects in a general setting where all features of the system are observed and controlled. To this end, we used an online prosthesis simulator (OPS) to validate our approach in experimental demonstrations [13]. In this system, an able-bodied user made overt movements which were detected and used to drive an artificial population of neurons (see methods for full details of our OPS). The simulated neurons were decoded in real time, enabling the user to control a cursor (via overt movements) in closed-loop, where the overall system performs analogously to a BCI. We present an example pipeline of the experiment in Fig 3—the user sees a static target and makes overt movements reflecting intention, the overt movements drive synthetic neural activity, and a cursor position is decoded from the synthetic activity. Here, the encoding model is considered the mapping from user intention (i.e. where the user wants the cursor to move) to noisy neural activity, and decoding goes from neural activity to cursor position.

If the appropriate signals are available, it is natural for a neuroprosthesis to use biomimetic control—for example, using the neurons which correspond to imagined left-right movement of the hands to control left-right movement of a cursor. With the OPS, we set out to explore a relevant regime where the user is inclined to use a biomimetic decoder (e.g. moving their hands naturally to control a cursor), but higher SNR is available in other, non-biomimetic dimensions which might be less likely to be explored. By deciding what body movements generate neural activity and how much noise is present for various neural channels, we can construct cases which require the user to learn in order to achieve optimal performance. Naïve motor-imitation control will not suffice because the native movement dimensions are noisier. Closed-loop learning is required in this setting so the user can learn the non-biomimetic scheme.

The purposes of these OPS demonstrations are (1) to examine how much performance might be expected to improve if optimal encoder-decoder pairs are used instead of sub-optimal, initial mappings and (2) to examine the extent to which non-biomimetic schemes are learnable. To this end, we compared the use of a pre-computed, fixed decoder for the cursor task with biomimetic schemes to illustrate the sense in which our approach finds better solutions. Given that we only receive signal from a small number of control dimensions (i.e. overt movement channels) and we wish to make some of these control dimensions noisy, we restricted attention to 1D cursor versions of the task. We compute the optimal decoder according to our framework and use it for the cursor task, expecting that the user should be able to learn it. As described when presenting our framework, this decoder corresponds to a theoretically obtainable end-result of a co-adaptation process having occurred, for which user learning was permitted.

#### OPS experimental results

After a few minutes of free exploration during which the user could become comfortable controlling the cursor (see methods for full description), subjects were evaluated on the “pinball” (point-to-point reaching) task. We compared subjects across two decoder conditions: whether the decoder was derived from motor-imitation initialization (described in methods) or using the jointly optimized, pre-computed decoding approach which follows from our framework. Each subject participated in three cases of the experiment, with each case differing in SNR level as well as how the overt movements mapped to neural signals (see methods). These three cases are different in details but are meant to provide multiple, independent demonstrations of the same effects.

**Fig 4:**
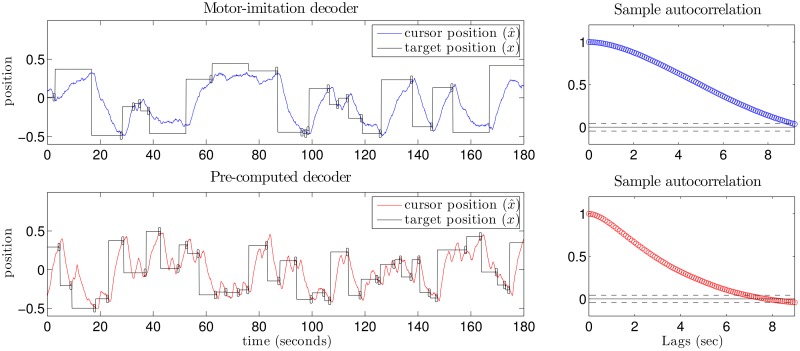
Comparison of the two decoding conditions for subject 2, SNR-case 3. The top plot depicts single trial data with a motor-imitation initialized decoder. The cursor moves more smoothly (i.e. oversmoothed) and it is difficult to reach extremally-located targets. This smoothness is reflected in a slow falloff for the autocorrelation function (ACF) of the cursor position. The bottom plot depicts a matched trial with a pre-computed decoder. The user’s better control derives from a decoder with comparable local smoothness, but more responsiveness. Empirically the ACF has correspondingly sharper falloff.

We first consider single-subject comparisons using each of the two classes of decoders. We observe that our method performs visibly better than the motor-imitation decoder (see Fig 4 for a representative example trial). In general, during motor-imitation decoder conditions, dynamic range of the cursor was limited (by noise, finite range of movement, and limb constraints) so subjects had difficulty reaching all extremal-located targets (see Fig 4). Weaker encoding leads to limited range of motion on screen and slower responsiveness on screen as a consequence of heavy smoothing/dampening, arising from relying more on the Kalman filter prior than the noisy observation. The level of smoothing is set automatically when optimizing the KF decoder to appropriately balance reliance on the prior and the encoding model. If the decoder places less weight on the prior, it does so because it has a sufficiently informative encoding model. Higher reliance on the prior indicates the use of a less informative encoding model, and less smoothing in this setting would have suboptimal error, permitting more jitter without compensating improvements in control. If encoding were stronger for noisier neural dimensions, the noise in those dimensions would be amplified, thereby undermining decoding. The timescale of smoothing can be empirically characterized by the autocorrelation function (ACF) of the cursor position when the user controls it, with slower autocorrelation falloff corresponding to slower, more sluggish control and heavier smoothing (see rightside panels of Fig 4). The timescale of smoothing in the decoder due to the prior arises in large part from the dominant eigenvalue of the *G* matrix of the decoder (which for a 1D setting is simply the value of *G*). Indeed, we find that *G* is larger for motor-imitation decoder conditions whenever the motor-imitation decoder ACF decays slower than the pre-computed decoder ACF (not shown).

**Fig 5:**
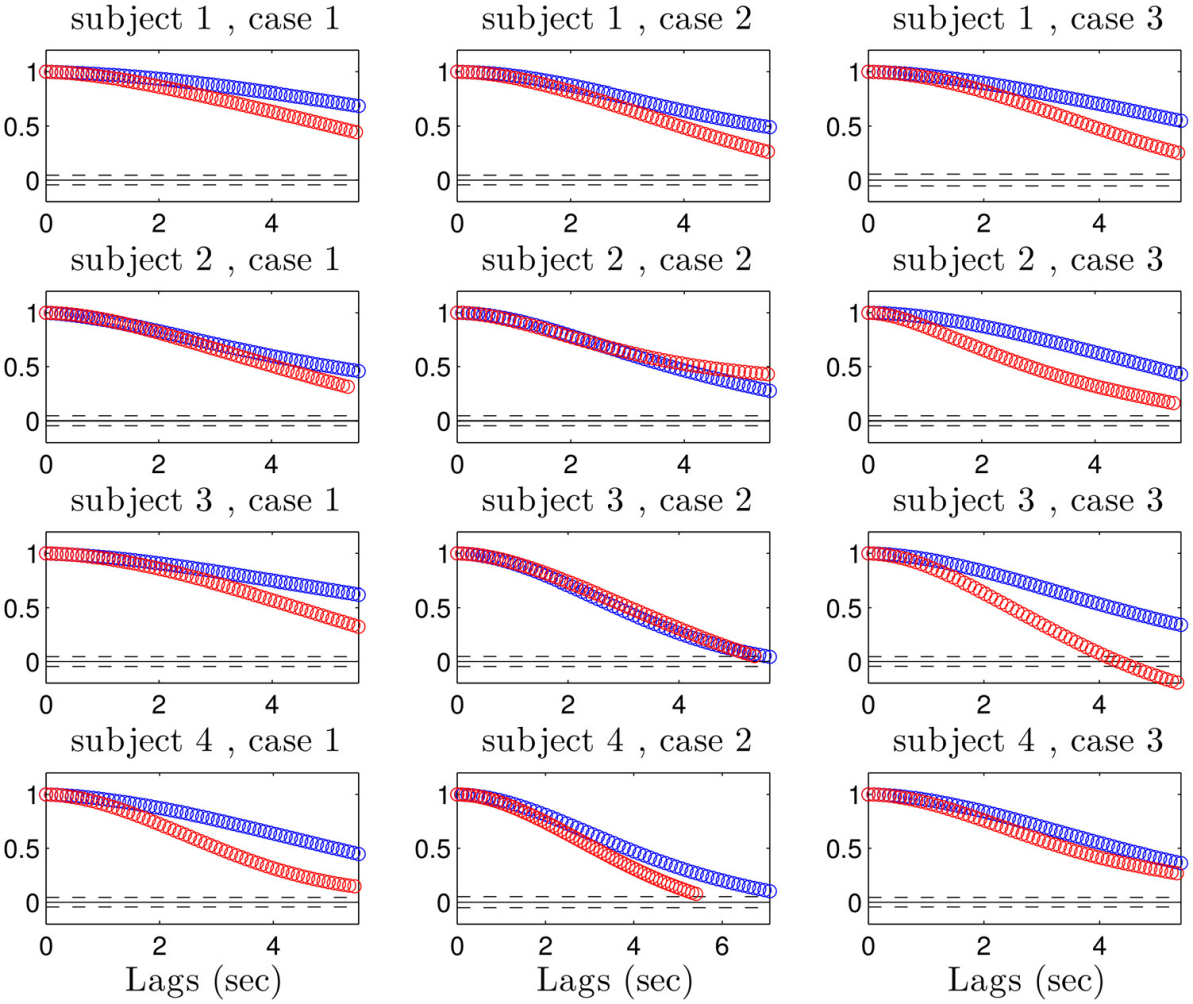
Cursor autocorrelation functions for all subjects and all conditions. For each SNR case, motor-imitation decoder curves are depicted in blue and the pre-computed decoder overlays it in red. Better controllability would be expected to be reflected in faster ACF falloff. In almost every case, falloff was faster for the cursor control using the pre-computed decoder (SNR-case 2 did not follow this trend as reliably—see main text for discussion).

**Fig 6:**
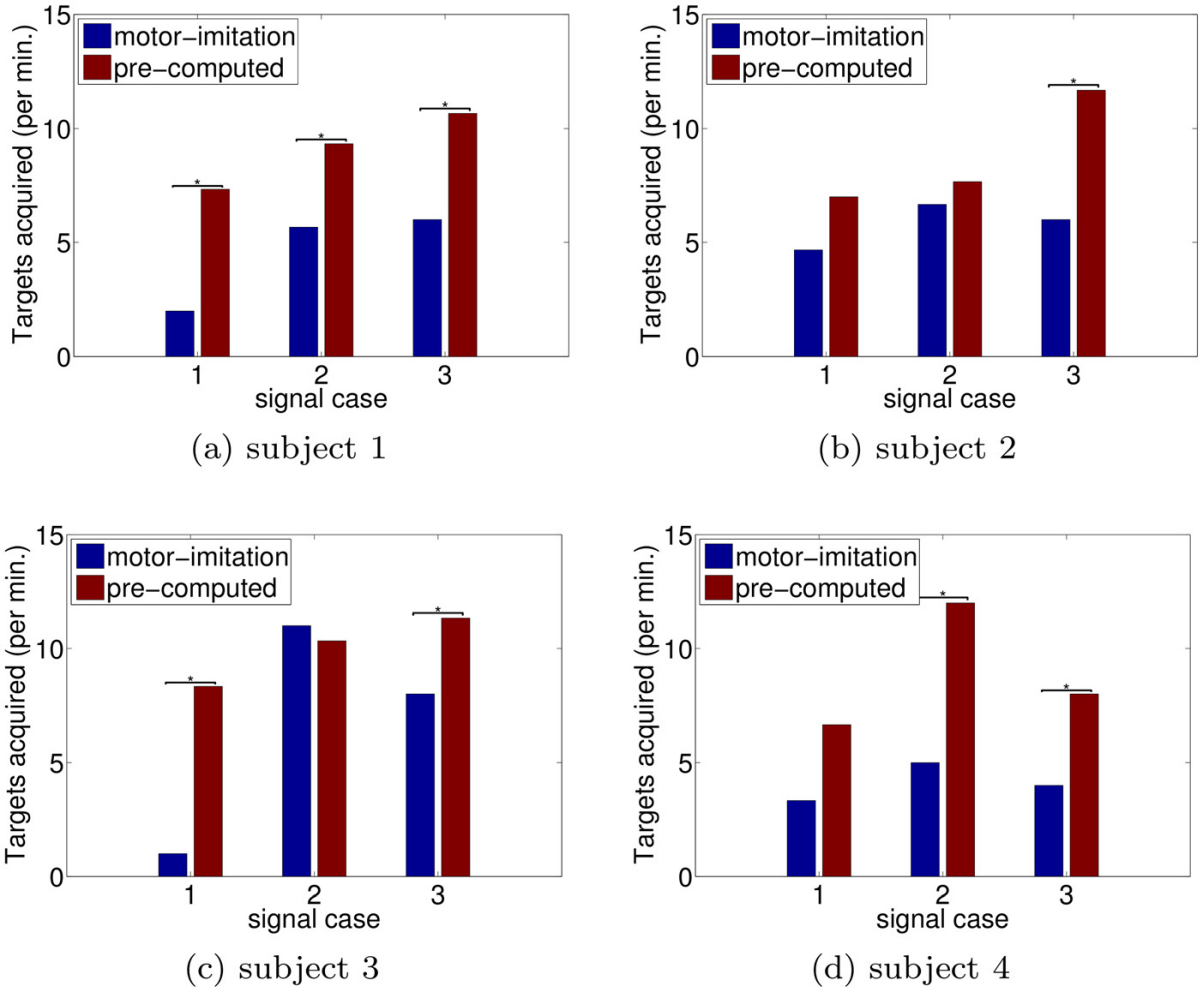
Each subfigure depicts performance comparisons for the different decoders in various SNR conditions for a particular subject. The quantity compared is the number of targets per time and and significance is tested with one-sided unpaired t-tests (*p* < .05) on time between acquisitions. In general, subjects tend to perform better on the task using the pre-computed decoders rather than those initialized by motor-imitation.

The details of the single-trial data shown in Fig 4 generalize to all three task conditions, across multiple subjects. Cursor ACFs across subjects and conditions are presented in Fig 5, with motor-imitation decoder conditions plotted in blue and pre-computed decoder conditions plotted on the same plots in red. The slower falloff in the ACF for motor-imitation decoder conditions indicates less controllability for this class of decoder. Moreover, when encoding uses low-SNR channels, the KF decoder has perceptible lag insofar as optimal decoding will rely more on the prior. This is reduced when using higher-SNR neural activity because the encoding model contributes more reliable information, which in turn requires less smoothing by the optimal decoder. For a given level of noise, there is necessarily a trade-off between responsiveness and posterior variance. Taken together, these features tended to make performance better when using the pre-computed decoder relative to the motor-imitation decoder as the former took better advantage of the available signal along less noisy dimensions (see Fig 6). Significance across decoders is tested with one-sided unpaired t-tests (*p* < .05) on time between acquisitions. In nearly all cases and across subjects, the performance of the pre-computed decoder dominates the performance using the motor-imitation scheme.

All three SNR cases were designed with the expectation that user “motor-imitation” behavior would consist of moving both hands together left or right to control a horizontal cursor (i.e. moving both hands rightwards to get the cursor to move rightwards, etc.). However, subjects 2 and 3 predominantly used one hand during SNR-case 2 motor-imitation trials, and this happened to correspond to a good non-biomimetic scheme—the distance between hands served as a signal which strongly drove neural activity. Consequently, the motor-imitation decoder had decent signal as these subjects’ motor-imitation preferences coincidentally aligned with high SNR dimensions. This meant that the pre-computed decoder did not have enough room to do meaningfully better. This is reflected both in the insignificant differences in performance (Fig 6) and also in the similar ACFs for case 2 across decoders (Fig 5).

**Fig 7:**
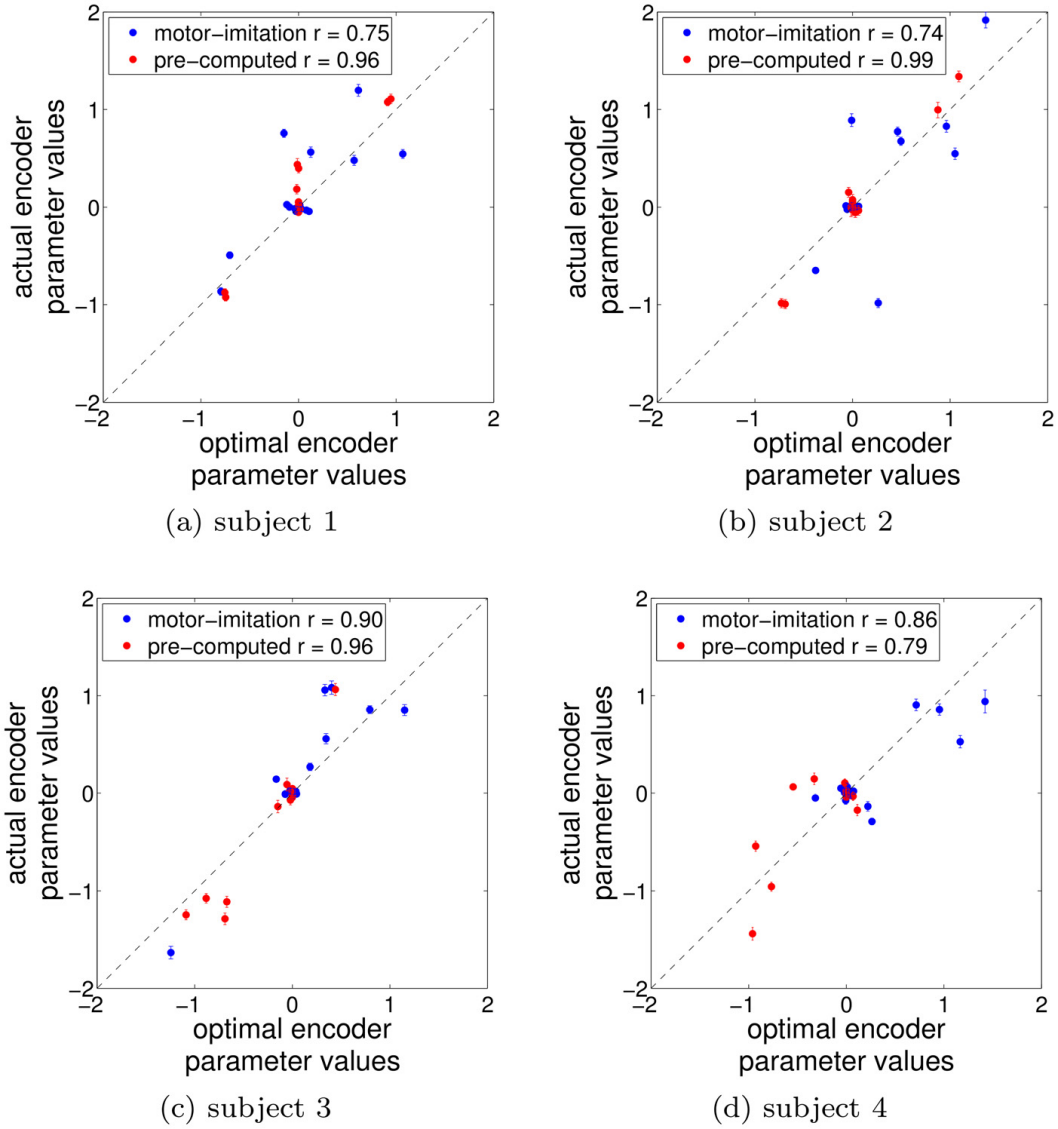
All subjects learned the coding schemes. Each plot compares, for each decoder, an estimate of the user’s learned encoder parameters against the optimal encoder parameters for a given subject, aggregated across signal cases. The optimal parameters for the two decoders are unrelated. A reference line is depicted on each plot and correlation coefficients are provided for each decoder (All correlations are significantly different from zero, *p* < < .01). Each point corresponds to a comparison between how much weight the user gave each channel vs how much weight the channel would have been given if optimally learned. Points near zero correspond to channels not heavily used. While perfect match would not be expected, the relatively strong correlations indicate that both decoder classes are learned reasonably well by all subjects.

In addition to the performance differences between decoders, we examined how well the subjects learned to control the cursor via the decoders, essentially to verify the learnability of the pre-computed decoder. To demonstrate this, we provide plots comparing the parameters of the optimal encoding models against the estimated actual encoding model of the user (Fig 7—see methods for details of estimation of encoding models). For each subject and decoder type, we compare the estimates of the subject’s encoding model parameters (how strongly the user relies on each neural channel) of all conditions against the optimal encoding model parameters for the decoder presented. The optimal encoder parameters for the motor-imitation and pre-computed decoders are not related and were in fact found to be quite distinct (data not shown). The purpose of this figure is merely to assess the extent to which each user was able to approximately match their encoder to the optimal encoding strategy. Relatively high correlations for both decoders for all subjects suggest that users have successfully learned an encoding scheme which approximately matches the decoder. This implies that differences in performance are related to the coding scheme and do not have to do with inadequate user learning. Effectively, for these examples using the OPS, the pre-computed decoder seemed to realize the theoretically expected performance improvements and these decoders seemed empirically quite learnable.

## Discussion

The key contributions of this paper are to establish co-adaptation methods as joint optimization problems over encoder-decoder pairs and to propose our approach for pre-computing an optimized decoder. For Kalman filter decoders and under our modeling assumptions, we showed that co-adaptation should not be able to do better than our approach in theory. To test the approach empirically, we also presented 1D OPS validation, in which our approach provided significantly better performance than the motor-imitation decoding approach. Instead of devising rules for adapting the decoder, we have presented an approach which characterizes what the user and decoder ought to do to complement one another, and we demonstrated that the user can match the optimal strategy in the 1D setting.

### Revisiting co-adaptation

Our framework points to some qualitative insights into co-adaptation. In presenting our framework, we pointed out a non-obvious property of KF-decoder updates—updates to the decoder that are designed to be optimal for the current encoding model can allow for improvement if the user learns further (i.e. adapts the encoding model). This is effectively why co-adaptation can serve as a coordinate descent algorithm. Implicitly, this property, coupled with the recognition that the user encoding can change, has permitted various approaches that attempt to track these changing encoders, using Kalman Filtering (e.g. CLDA [4]) as well as reinforcement learning (e.g RLBMI [9]). In both settings, the decoder is adapted in a principled fashion which permits tracking as a user adapts, so user and decoder collaboratively improve task performance (via a coordinate-descent-like method). These approaches may adapt the decoder slowly over many days. However, there is an intrinsic trade-off between lag and noise when using adaptive methods—the decoder can either adapt slowly when it has enough information to make good steps or adapt quickly but be highly susceptible to noise in the updates [33]. Slow updating induces delays in decoder adaptation which may make exploration by the user difficult because the user may attempt to explore on timescales shorter than the lag.

We can contextualize our pre-computation approach relative to standard co-adaptive methods, by viewing pre-computed decoders as an attempt to push towards a certain logical extreme, essentially with the decoder “adapting” entirely before any user learning takes place. Leveraging available information about the statistics of the recorded signals, the decoder can change pro-actively to prompt the user, who may not be learning very efficiently, to move in a direction in which better performance is expected. This is effectively what our algorithm was designed to do. A core intuition in assessing the relationship between these approaches is the observation that if one dimension of neural activity has less noise than another, optimal decoding will rely more heavily on the less noisy dimension. If noisy and non-noisy dimensions initially have non-zero weight, perhaps co-adaptation can be used to tune the relative strengths. However, this tuning can also be computed directly given estimates of signal and noise statistics. We believe our framework helps clarify the performance opportunities available when using fixed or adaptive decoders.

### Relationships to other approaches

Our approach is broadly consistent with the literature on joint source-channel coding, but we are not aware of neural modeling being performed using our formulation. In the control literature, others have derived solutions to the related problem of optimizing time-varying observation models for the time-varying Kalman filter, but their result does not extend to the steady state case and concludes with a numerical analysis showing that their solution can be very suboptimal for our setting [34].

There also exists other research which has looked into control schemes for “body-machine interfaces”, for example using a sensor-wired glove with many degrees of freedom (DOF) to control a cursor-on-a-screen [35–37]. This research is generally consistent with our approach, and also uses the term co-adaptation in a way which refers to situations where a user learning over time can be complemented by the decoder adapting to the new encoding of the user. The body-machine interface literature discusses various options for selecting the encoder, but they do not model the effects of noise or the relative differences between different encoder choices. One approach uses principal component analysis (PCA) on calibration data and then maps the first *n* principal components to the *n* effector DOFs by an arbitrary mapping which can be relatively straightforward for the user to then learn by exploration [38, 39]. Our approach resembles the PCA approach if noise and temporal correlations are ignored, since we also rely on unsupervised collection of second order statistics. However, neural noise plays a significant role for us. Consider that in our 1D demo the total covariance has high variance in noisy dimensions. A naïve application of the PCA-based decoding scheme will result in a decoder which most heavily relies on the noisiest dimensions simply because they have more variance. Alternatively, when an adaptive decoder is used, the adaptation proceeds from a reasonable initialization [36, 37]—for the neural case, our approach helps find a reasonable scheme without depending upon any well chosen initialization.

### Further considerations

There are a few points to be aware of when interpreting the results of this paper. We optimize encoder-decoder pairs for the target tracing task, and we rely on similarity between tracing and the pinball tasks for the derived optimal solutions to hold in the pinball case. Additionally, we still have a free parameter in our optimization, *λ*, for tuning the magnitude of the neural activity constraints. For OPS experiments, we did not optimize performance over *λ*; rather, we tuned it very crudely to the parameters of the synthetic neural activity and held it fixed across subjects.

A limitation of the present study is that learning in an OPS setting may be different from learning in real BCI or across classes of BCI [40, 41]—for example, myoelectric interfaces engage later stages of a motor hierarchy, so timescales and constraints of learning may be different [40]. Consequently the extent of learnability and timescale of learning of our fixed, optimized decoder may vary by setting (OPS vs BCI type). While the OPS may serve as a relevant proxy of BCI in many respects [13], ultimately experiments in specific settings will be required to test the utility of our approach to each.

That said, both the decoding approach and the OPS can be modified to incorporate other features of the neural activity such as neural dynamics. While not conventionally modeled in BCI, recent research has revealed dynamical components to neural activity in motor cortices [42]. For such a class of neural activity used for the neuroprosthesis, one could consider the extensions $y_{t}=Ax_{t}+B{\hat{x}}_{t-1}+Dy_{t-1}+noise$ as a model of the neural activity. Moreover, although we used kinematic signals as the inputs to our simulated neurons, other user-controllable signals (e.g. muscle activity) could be used as inputs into the encoding model, possibly affecting the results. The inputs to the OPS are merely intended to serve as reflections of user intention and not necessarily what the real neural activity encodes in normal, non-BCI behavior.

We lastly emphasize that our methods require estimation of the neural noise covariance, so a calibration session used to examine neural activity is of crucial importance. For real neural applications, the neural signal and noise components could be estimated using various neural factor analysis methods [43, 44].

### Future directions

We have made an initial attempt to build learnable decoders using a new joint optimization framework. We have focused on the linear-Gaussian setting which is implicit when KF decoders are employed. There will certainly be model mismatch, and insofar as this model proves too simple, the way forward is to incorporate additional features of the encoding model and constraints on user learning. While our approach is very clear about the extent to which it relies upon the accuracy of these assumptions, it is worth emphasizing that co-adaptation approaches are equally (if implicitly) dependent on these assumptions. For example, the structure and timescale of decoder updates in co-adaptive approaches directly impact how well users can learn [36] or whether the user learns at all [5]. By making these assumptions explicit, we hope that we can improve the adaptive decoder engineering process.

Additionally, while KF decoders are still widely used in BCI, future improvements may require nonlinear decoding methods, so we may wish to extend our framework to certain classes of nonlinear encoder-decoder pairs. For example we could consider Poisson-observation encoding with point-process decoders or otherwise parameterize the neural noise covariance to scale with the magnitude of the signal (i.e. signal-dependent noise as in [45]). Generally, any processes (even discrete ones) could be specified for *x* and *y*. Following the conceptual approach proposed here, the objective function over encoder and decoder parameters could be specified and optimized subject to constraints. Furthermore, a full decoding system would certainly incorporate additional components such as modifications for increased stopping reliability [46] and approaches for longer-timescale nonstationarity tracking as in [47, 48].

In our OPS experiments the modeling assumptions hold by construction, so how significantly results will deviate from those of this paper in physiological experiments is open. Also, we admittedly focused on low dimensional control in order to gain conceptual ground, but this is simpler than high dimensional control. It remains to be explored how much more difficult user learning is in high dimensional settings, and what modifications will be required to assist users in learning optimal encodings. Additionally, more detailed characterization of the learning process may become relevant.

If applying our framework to human BCI, there are a number of practical opportunities. For example, it may be interesting to develop interfaces which indicate to the user how much control is available for some number of DOF and permit the user to select how to map control to the BCI effector. Users may have specific preferences to invert certain dimensions of control or request more precision on a certain effector DOF even at the expense of precision for other DOF. Such user-specific modifications are entirely amenable within our approach.

## Materials and Methods

All simulations, optimizations, and experiments were conducted using custom code in Matlab (**see corresponding author’s website for code implementing the main algorithm**). As noted when presented in the text, full derivations of our methods are provided in S1 Text. The remainder of this methods section describes the experimental methods.

### Ethics statement

Human protocols were approved by the Columbia University Morningside Institutional Review Board—all subjects read an IRB-approved informed consent document and provided verbal consent (Protocol Number: IRB-AAAM6407).

### Online prosthesis simulator as a model of BCI

**Fig 8:**
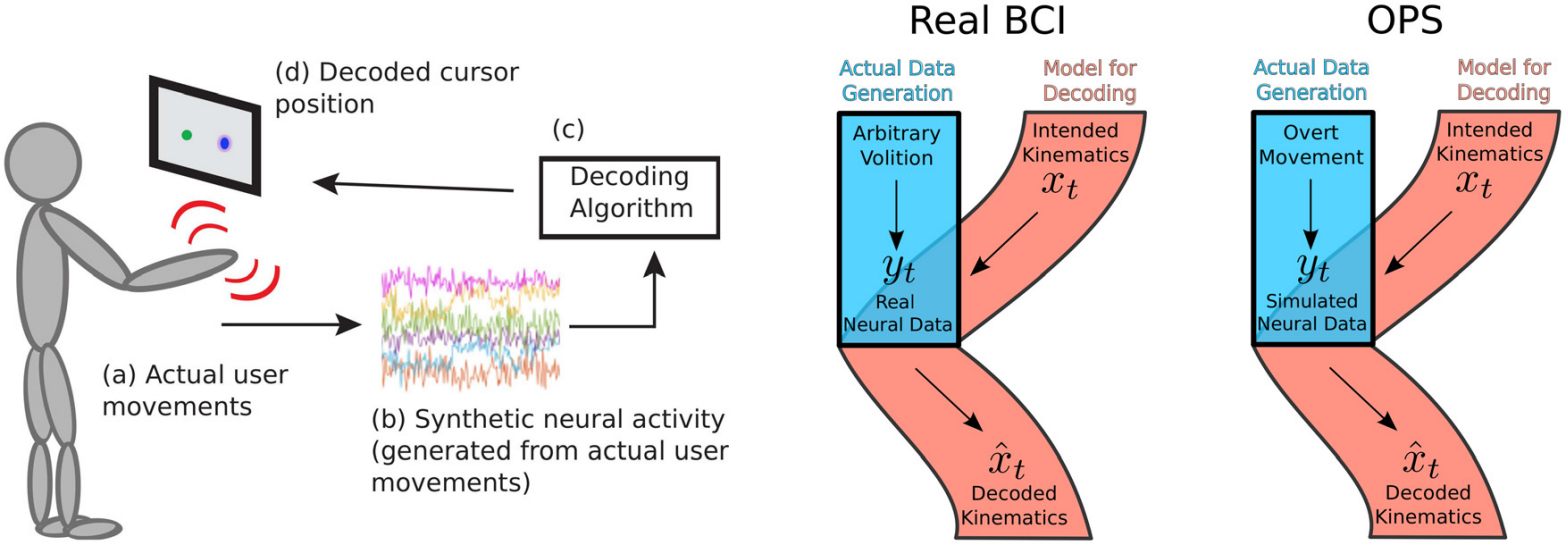
OPS setup is depicted and related to real BCI. The cartoon on the left depicts the OPS: (a) Actual overt movements of the user are detected. (b) Synthetic neural activity is generated in some fashion derived from the overt movements. (c) The decoder sees only the simulated activity and provides a decoded estimate of the cursor position. (d) The cursor is placed on the screen along with the target permitting visual feedback. The flow diagrams on the right compare how data is generated in a real BCI versus the OPS and the relationship between the variables. While there is no direct link relating intended kinematics and overt movement (or volition), these variables are conditionally related. If the user desires to produce a certain decoded kinematics and doing so requires some modulations of the neural activity which are controlled by the overt movement, then the user should modulate overt movement in a way that reflects their intended kinematics.

The Online prosthesis simulator (OPS) is an experimental platform that tracks overt movements of the user, uses the movements to simulate noisy neural data, and then decodes the synthetic data by a brain-computer interface (BCI) decoding algorithm [5, 13]. We captured the overt movements of able-bodied users with a Microsoft Kinect. The Kinect interfaces to a standard computer via usb and signals were analyzed in real time in matlab (middleware for Kinect-to-Matlab follows [5]). Kinect data for our experiments was collected at approximately 10Hz. Movement data was used to simulate neural signals by an arbitrary scheme, selectable by the experimenter—we chose to generate neural activity by de-meaning the movement signals, linearly mixing them, and adding independent Gaussian noise to each neural channel. This simulated neural data was then analyzed by real-time BCI algorithms during experiments. The decoded results of the simulated data were presented as feedback to the user so the user could change their movements. See Fig 8 for a visual depiction of this system.

The user’s kinematic intention (*x*_*t*_) should correspond to an attempt to control the cursor towards a target presented on the screen. The overt arm movements of the user are used to simulate a population of synthetic, noisy neural units (*y*_*t*_). The encoding model (*A*) is the estimated linear mapping from *x*_*t*_ to *y*_*t*_. During the task, the neural activity is combined with the decoded estimate of the previous timestep (${\hat{x}}_{t-1}$) to determine the current cursor position (${\hat{x}}_{t}$). The decoded cursor position comes from the use of the optimal steady state Kalman filter (using *F* and *G* via Eq 3).

In some high level sense, the user could explore the space of mappings from their behavior to visible feedback. While the user could not change how their overt movements map to neural activity, the user could change the relationship between “kinematic intention” and simulated neural activity—this is the relationship we call the encoding model. That is, changes in how the user made overt movements when intending to obtain certain decoded kinematics will cause changes in the encoding model (estimated parameters *A*).

We emphasize that these overt movements are not supposed to correspond to overt movements in a BCI setting; rather, these overt movements are simply supposed to provide arbitrary volitional signals which the BCI system will exploit to perform a task. BCI permits the use of any neural signals which are correlated with user volition—for example, even imagined speech has been used to control a 1D cursor [49]. The OPS could be driven with arbitrary control signals, and overt movement signals seem a reasonable model of the class of signals which may in part be used to drive a real BCI.

### Experimental procedures

To analyze optimal encoder-decoder pairs, we have been considering the objective function of a “tracing” task (a.k.a. pursuit tracking), in which the user has a time-varying kinematic intention and the decoder attempts to optimally estimate the kinematics. This objective is standard, physically meaningful, and the methods developed from it yield smooth priors. We validated our methods using the OPS on a “pinball” task (a.k.a. point-to-point reaching) where the user controls a smoothly moving cursor towards targets, as this is more realistic for applications.

In this work, we present the results of a 1D pinball task. We present data from 4 subjects (none of whom are authors of the study or otherwise directly affiliated with this project). Subjects were explained the rules of the “game” they were to play but were not described details of simulated neural activity (some had performed prototype versions of the experiment previous to participation in the experiments reported here). Before beginning the task, we collected calibration data for each user—the user was instructed to move around freely in an unstructured way. During this time, the user saw real-time video (RGB image frames) of themselves with skeletal tracking superimposed, in order to see how well the Kinect tracking worked. This unstructured phase was to get an estimate of the empirical neural activity covariance (i.e. estimated as the sample covariance of the neural responses).

The subjects participated in one session for each of two decoders (“motor-imitation” and “pre-computed”, described more below) in each of three “signal cases”. Signal cases consisted of specifications of how strongly the simulated neural channels were driven by different tracked body movements of the user (described more below). Between each of these six conditions, subjects were given a break until they felt ready for the next. For each signal case, the two decoder sessions were presented with the motor-imitation trial first, followed by a trial where the cursor was controlled by the pre-computed decoder. These trials were independent and the decoders were unrelated so the order mattered only so the control strategy the user learned when using the pre-computed decoder would not affect the motor-imitation control scheme. For block-order validation see S1 Fig.

Each session consisted of two key phases, “free exploration” and “testing.” During free exploration, the user controlled a cursor on a screen via closed-loop OPS control, and there was no target. During this time, the user was prompted to explore and try to learn as well as possible how to control the cursor—this phase gave the subject an opportunity to learn, and nothing about the decoder was changed. Exploration rapidly provided information about how the user’s intentions relate to the movement of the cursor. The user can adjust their control strategy, in turn, adjusting the tuning of the simulated neurons—this process constitutes learning in this task. After a few minutes of exploration, a target appeared and the user was prompted verbally to move to the testing phase when ready by holding the cursor at the target for a few seconds (subject 1 moved directly to the testing phase without having to hold a target first, and this subject reported having had adequate time in free exploration). During testing, a single target at a time was presented, surrounded by a visible halo. The user attempted to control their cursor to the target and attempted to “acquire” the target by holding the cursor in the halo-region (holding is indicated by a color change of the region inside the halo). After a short period of time, if the cursor is held within the halo (≈1s), the target is acquired, disappears, and a new target is randomly placed at a new location. Targets that were not acquired after ≈15s were treated as misses, and a new target replaced them. The subject was instructed to acquire as many targets as possible in the ≈3min duration of the testing phase. Before beginning the task, the entire trial structure was verbally described to each subject, and verbal reminders of trial-stage (at transitions) were provided by the experimenter. A video depicting the experimental procedures is included as S1 Movie.

### Decoders

The two decoders tested were selected either by “motor-imitation” initialization or by pre-computing the optimal encoder-decoder pair and presenting the optimal decoder. Trials with “motor-imitation” decoders began with an extra phase (at the beginning of the free exploration phase), during which a target moved smoothly on the screen. The user was instructed to “trace” the target with their hand movements, thereby providing the “native” control mapping of making rightwards and leftwards movements of their hands to control a cursor along the horizontal axis (without visual feedback, i.e. open-loop). This OPS phase provided supervised data which permitted estimation (by recursive least squares—an online algorithm for linear regression) of a motor-imitation encoding model, a control scheme which is as close as possible to user default control. The motor-imitation decoder refers to the optimal decoder corresponding to this encoding model, which can be computed directly from the estimated encoding model. Alternatively, for the pre-computed decoder trials, we used covariance information determined from the calibration data to estimate an optimal encoder-decoder pair according to our joint optimization approach presented in this paper, and we presented the pre-computed optimal decoder to the user. This second approach does not require labeled data; rather it merely optimizes the encoder-decoder pair such that good task performance results after the user learns how to use the scheme. Selection of the decoder is unsupervised, and this approach relies on the user to adapt to match the fixed decoder.

We note that *P* and *Q* (i.e. the prior dynamics parameters of Eq 1) are the same for both cases, with *P* selected just under 1 (i.e. .99) to discourage drift and *Q* selected to be small (.01), which was determined by the sample rate and the distances in the task to permit appropriate movement rates for this task. Strictly, *Q* is a free parameter which affects smoothing, but it should be selected in a task-dependent manner matched to the speed of target movement (or distance between targets).

### Signal cases

Using the OPS, we are able to arbitrarily specify mappings of user movements to the simulated neural data as well as setting noise levels across channels—this corresponds to the blue box of the OPS flow diagram in Fig 8. To compare results across subjects, we selected three pre-defined mappings by which overt movement would drive simulated neural channels (k = 6) along with corresponding neural noise structure. These could be thought of either as a small number of electrodes or pre-processed neural data (e.g. after dimensionality reduction). Noise is the same order of magnitude as the signal—this is realistic for single units. For each channel, noise is set to a fixed high or low value, with high noise set to be approximately the same magnitude as the signal of a heavily used channel and low noise set to be less than the signal power of a heavily used channel (per channel). Optimal encoder-decoder pairs necessarily take into account user calibration data so it is not possible to know in advance precisely what the optimal encoder-decoder pair will be. With that said, the optimal coding scheme should leverage the highest SNR dimensions. For comparison, the motor-imitation scheme which the user initializes will consist of rightwards and leftwards movements to control the cursor rightwards and leftwards respectively.

*Case 1:* Simulated neural activity is linearly modulated by positions of the right hand in horizontal and vertical dimensions—one channel driven by horizontal movement, another by vertical movement. Noise is low in the vertical dimension channel and high in the horizontal dimension channel, so the optimal encoder-decoder pair will predominantly encode the movement axis in the y movements of the right hand.

*Case 2:* Simulated neural activity is linearly modulated by horizontal position of both hands together as well as distance between hands—one channel driven by the sum of the two hands’ horizontal position, another channel by their difference. The channel driven by distance between hands has low noise so the optimal encoder-decoder pair will predominantly encode the movement axis in channel corresponding to the distance between hands.

*Case 3:* Simulated neural activity is linearly modulated by positions of each hand in horizontal and vertical dimensions independently—separate channels are driven by each of these variables. Noise is low on the simulated activity driven by vertical dimension of each hand, so subjects should move both hands together vertically.

We compared all subjects on all three cases of signal-noise structure using both motor-imitation initialized decoders and pre-computed decoders. The above cases were selected with the intent of highlighting differences between naïve, motor-imitation schemes and schemes which permit better performance by coding in dimensions that are not natively leveraged. The intention was to see if optimization of the decoder yielded performance differences and how learnable the schemes were.

### Estimation of encoding models

In the results, we made use of estimated user encoding models, comparing them with optimal encoding models. To estimate the user encoding models during the pinball task, we simply performed linear regression from target positions to neural activity during the pinball phase. This assumes that the user is attempting to control the cursor such that it moves directly towards the target.

## Supporting Information

S1 Text Mathematical appendices including detailed derivations of methods used for joint encoder-decoder optimization. (PDF)

S1 Movie “Simple demonstration of experimental procedures”. Side-by-side video of tracked user pose along with cursor control task. For high SNR, user initializes the decoder by motor-imitation, freely explores to become comfortable with the scheme, and then proceeds to the testing phase. The data used in the video was collected for demonstration purposes only.
(MOV)

S1 Fig “Block order validation”. Two additional subjects were run for alternating blocks of testing with the motor-imitation and pre-computed decoder on signal case 1 (three tests blocks using each decoder per subject). The initialization for the motor-imitation decoder was run once at the beginning. Between decoder switches, subjects were given short exploration phases to refamiliarize themselves before testing. One subject began with the motor-imitation decoder, and the other began with the pre-computed decoder. Performance across subjects and blocks was significantly better using the pre-computed decoder (using a one-sided t-test on targets/time across repeats and subjects). Across subjects and repeats, no statistically significant linear trend was found for performance with the motor-imitation decoder. A weak trend was found for the pre-computed decoder across repeats (i.e. for these subjects there was some evidence that subjects continued to improve when using the pre-computed decoder *p* < .05)
(TIFF)
