# Supplementary material for: Encoder-Decoder Optimization for Brain-Computer Interfaces
Source: PLoS Comput Biol. 2015 Jun 1;11(6):e1004288. doi: 10.1371/journal.pcbi.1004288 (PMC4451011; doi:10.1371/journal.pcbi.1004288)
Supplement: S1 Text — (PDF) [file pcbi.1004288.s001.pdf]

Supporting Text – Appendices

A Encoder-decoder optimization for i.i.d Gaussian input

Here we present a derivation of the optimization routine for the optimal encoder-decoder pair when the input distribution is i.i.d. Gaussian. This problem is essentially the same as the core problem presented in Doi et al. (2007) – we present the solution here for a general covariance structure. If the input x is actually drawn from an AR(1) process, we could consider only the marginal statistics and this encoder-decoder solution would be optimal for the class of decoders without a prior model.

Using the linear decoder, consider that

$$\begin{aligned} \hat{x}_t - x_t &= FAx_t + F\epsilon_t - x_t && (1) \\ &= (FA - I)x_t + F\epsilon_t && (2) \end{aligned}$$

$$\begin{aligned} \mathcal{E}(A, F) = \mathbb{E}[(\hat{x}_t - x_t)^T(\hat{x}_t - x_t)] &= tr(\mathbb{E}[(\hat{x}_t - x_t)(\hat{x}_t - x_t)^T]) && (3) \\ &= tr(\mathbb{E}[(FA - I)x_t x_t^T (FA - I)^T + 2(FA - I)x_t\epsilon_t^T F^T + F\epsilon_t\epsilon_t^T F^T]) && (4) \\ &= tr((FA - I)\Sigma_x(FA - I)^T) + tr(FCF^T) && (5) \end{aligned}$$

$\mathcal{E}(A, F)$ is our objective function and has free parameters for the encoder and decoder. In this formulation, we want to first try to solve for the decoder. Things work out nicely because we can solve for the decoder in closed form in terms of the encoder. We then plug this back into the overall objective function which now is in terms of encoder parameters and then we can optimize this objective. We use techniques from matrix calculus to solve this (for reference, see Petersen and Pedersen (2012)).

$$\begin{aligned} \frac{\partial \mathcal{E}(A, F)}{\partial F} &= \frac{\partial}{\partial F}[tr((FA - I)\Sigma_x(FA - I)^T) + tr(FCF^T)] && (6) \\ &= -2\Sigma_x A^T + 2FA\Sigma_x A^T + 2CF^T && (7) \\ (\text{set} = 0) & && (8) \\ \Sigma_x A^T &= F(A\Sigma_x A^T + C) && (9) \\ F &= \Sigma_x A^T(A\Sigma_x A^T + C)^{-1} && (10) \end{aligned}$$

We incorporate the SNR penalty, $\mathcal{G}_{SNR}(A)$ (from the main text), by adding it to the objective. It only enters into the gradient-based optimization of A, so it is fully incorporated by adding the appropriate gradient to the gradient of the MSE objective when computing the gradient with respect to A. The gradient of $\mathcal{G}_{SNR}(A)$ with respect to A is:

$$\frac{\partial}{\partial A}\mathcal{G}_{SNR}(A) = C^{-1}A\Sigma_x^T + C^{-1}A\Sigma_x = 2C^{-1}A\Sigma_x \propto \lambda C^{-1}A\Sigma_x \quad (11)$$

using some appropriately chosen λ to enforce a particular SNR constraint. Taken together, the update rule for A is given by $A \leftarrow A + \eta\Delta A$, where η is a small, fixed stepsize and the step-direction ΔA is given by:

$$\Delta A = -F\Sigma_x(I_n - FA)^T - \lambda C^{-1}A\Sigma_x \quad (12)$$

We can consider this algorithm for robust coding without a AR prior to be a sort of baseline against which the other methods ought to be compared - obviously a fair comparison requires limiting the SNR comparably.

Note that in this section, the covariance Σ_x is the marginal covariance of the variable x. If x is actually an i.i.d. process, this is simply the covariance, however we use Σ_x in the main paper to refer to the marginal covariance of the AR(1) process ($\Sigma_x = P\Sigma_x P^T + Q$). Σ_x here corresponds to the AR(1) marginal covariance, so as $P \to 0$, then $\Sigma_x \to Q$.

B Encoder-decoder optimization for AR(1) input

In this section, we show how to optimize the encoding model using a gradient rule on the objective function for the AR(1) source (i.e. a gradient rule for updating A). This would serve as a step in an algorithm to find the optimal encoder-decoder pair. In this section, $G = P - FAP$ is always assumed, since this is the requirement for unbiased Kalman Filter estimates.

B.1 Notation and Preliminary Results

It is useful to state some results for Kalman filters. First we define the prediction as

$$\hat{x}_{t+1|t} = \mathrm{E}[x_{t+1}|y_{1:t}] = \mathrm{E}[Px_t + z_{t+1}|y_{1:t}] = P\mathrm{E}[x_t|y_{1:t}] = P\hat{x}_{t|t} \tag{13}$$

The associated prediction covariance for this estimate is given by

$$\begin{aligned}
\Sigma_{t+1|t} &= \mathrm{E}[(x_{t+1} - \hat{x}_{t+1|t})(x_{t+1} - \hat{x}_{t+1|t})^T|y_{1:t}] & (14)\\
&= \mathrm{E}[(Px_t + z_{t+1} - P\hat{x}_{t|t})(Px_t + z_{t+1} - P\hat{x}_{t|t})^T|y_{1:t}] & (15)\\
&= P\mathrm{E}[(x_t - \hat{x}_{t|t})(x_t - \hat{x}_{t|t})^T]P^T + 2P\mathrm{E}[(x_t - \hat{x}_{t|t})z_{t+1}^T] + \mathrm{E}[z_{t+1}z_{t+1}^T] & (16)\\
&= P\Sigma_{t|t}P^T + Q & (17)
\end{aligned}$$

where the last line comes from defining the posterior covariance, $\Sigma_{t|t} = \mathrm{E}[(x_t - \hat{x}_{t|t})(x_t - \hat{x}_{t|t})^T]$, and recognizing that $\mathrm{E}[(x_t - \hat{x}_{t|t})z_{t+1}^T] = 0$. In steady state we will denote the prediction covariance as, Σ_{SS}. It satisfies the following steady state Riccati equation

$$\Sigma_{\mathrm{SS}} = P(\Sigma_{\mathrm{SS}} - \Sigma_{\mathrm{SS}}A^T(A\Sigma_{\mathrm{SS}}A^T + C)^{-1}A\Sigma_{\mathrm{SS}})P^T + Q \tag{18}$$

Note that this equation has no dependence on F. Standard results (see *e.g.* Lancaster and Rodman (1995)) allow the Riccati equation to be rewritten as

$$\Sigma_{\mathrm{SS}} = P\Sigma_{\mathrm{SS}}\left(I + A^TC^{-1}A\Sigma_{\mathrm{SS}}\right)^{-1}P^T + Q \tag{19}$$

This equation facilitates the calculation of derivatives later.

The last result on Kalman filters is for the posterior covariance. Observing that we can write

$$\hat{x}_{t|t} = \hat{x}_{t|t-1} + F(y_t - A\hat{x}_{t|t-1}) = \hat{x}_{t|t-1} + FAx_t - FA\hat{x}_{t|t-1} + F\epsilon_t \tag{20}$$

we have

$$x_t - \hat{x}_{t|t} = (I - FA)(x_t - \hat{x}_{t|t-1}) - F\epsilon_t \tag{21}$$

which allows us to write

$$\begin{aligned}
\Sigma_{t|t} &= \mathrm{E}[(x_t - \hat{x}_{t|t})(x_t - \hat{x}_{t|t})^T|y_{1:t}] & (22)\\
&= \mathrm{E}[\left((I - FA)(x_t - \hat{x}_{t|t-1}) - F\epsilon_t\right)\left((I - FA)(x_t - \hat{x}_{t|t-1}) - F\epsilon_t\right)^T] & (23)\\
&= (I - FA)\Sigma_{t|t-1}(I - FA)^T + FCF^T - 2(I - FA)\mathrm{E}[(x_t - \hat{x}_{t|t-1})\epsilon_t^T]F^T & (24)\\
&= (I - FA)\Sigma_{t|t-1}(I - FA)^T + FCF^T & (25)
\end{aligned}$$

In steady state, we will denote this Kalman posterior covariance by Σ_{KP}. The objective function can be written as

$$\begin{aligned}
\mathcal{E}(A, F) &= \mathbb{E}[(\hat{x}_t - x_t)^T(\hat{x}_t - x_t)] & (26)\\
&= tr(\mathbb{E}[(\hat{x}_t - x_t)(\hat{x}_t - x_t)^T]) & (27)\\
&= tr(\Sigma_{KP}) & (28)
\end{aligned}$$

B.2 Minimizing the posterior variance

To minimize the KF posterior variance, we minimize the trace of the covariance of the current state estimate, given by Eq. (25), with respect to F and A, subject to regularization on A. We ignore regularization, taking for granted that the gradient of the $\mathcal{G}_{SNR}(A)$ with respect to A should be incorporated into the updates as in appendix A.

$$\mathcal{E}(F, A) = \mathrm{E}[\|x_t - \hat{x}_{t|t}\|^2] \tag{29}$$

$$= \mathrm{tr}\left(\mathrm{E}\left[(x_t - \hat{x}_{t|t})(x_t - \hat{x}_{t|t})^T\right]\right) \tag{30}$$

$$= \mathrm{tr}\left((I - FA)\Sigma_{\mathrm{SS}}(I - FA)^T + FCF^T\right) \tag{31}$$

where the final step uses the definition of the prediction covariance in Eq. (14) and the result in Eq. (25) for the posterior covariance.

Taking the derivative of Eq. (31) with respect to F, recalling from Eq. (18) that Σ_{SS} does not depend on F, and setting it to zero yields the standard Kalman state estimator

$$F = \Sigma_{\mathrm{SS}} A^T \left[A\Sigma_{\mathrm{SS}}A^T + C\right]^{-1} \tag{32}$$

In order to calculate the derivative of Eq. (31) with respect to A, one must take into account that Σ_{SS} is a function of A. In Konstantinov et al. (1993), they calculate the differential of Σ_{SS} in responses to changes in A. They make use of the closed loop system matrix

$$A_c = \left(I_n + A^T C^{-1} A \Sigma_{\mathrm{SS}}\right)^{-1} P^T \tag{33}$$

in order to concisely express the differential, $\partial_A \Sigma_{\mathrm{SS}}$, in response to changes in A as a solution to

$$\partial_A \Sigma_{\mathrm{SS}} - A_c^T \partial_A \Sigma_{\mathrm{SS}} A_c = -A_c^T \Sigma_{\mathrm{SS}} \partial_A (A^T C^{-1} A) \Sigma_{\mathrm{SS}} A_c \tag{34}$$

Straightforward manipulations allow the calculation of $\partial \mathrm{vec}(\Sigma_{\mathrm{SS}})/\partial \mathrm{vec}(A) \equiv D_A \Sigma_{\mathrm{SS}}$ from

$$(I_{n^2} - A_c^T \otimes A_c^T)\partial_A \mathrm{vec}(\Sigma_{\mathrm{SS}}) = -(I_{n^2} + K_{nn})\left((A_c^T \Sigma_{\mathrm{SS}}) \otimes (A_c^T \Sigma_{\mathrm{SS}} A^T C^{-1})\right)\partial_A \mathrm{vec}(A) \tag{35}$$

where $\otimes$ is the Kronecker product. Finally, the derivative with respect to A can be written

$$\frac{\partial \mathcal{E}(A, F)}{\partial A} = -2F^T(I_n - FA)\Sigma_{\mathrm{SS}} + \mathrm{vec}_{k,n}^{-1}\left\{\mathrm{vec}\left((I_n - FA)^T(I_n - FA)\right)^T D_A \Sigma_{\mathrm{SS}}\right\} \tag{36}$$

We optimize the encoder and decoder jointly via a coordinate-wise algorithm on the objective – we alternate between updating A via a gradient-based method (with appropriate regularization) and computing the optimal F,G parameters.

B.3 Relationship between AR(1) and i.i.d input solutions

We would like to conclude by verifying that the solution to the full problem appropriately generalizes and outperforms the marginal solution from appendix A. In the limit of $P \to 0$ which eliminates the temporal dependencies, we wish to confirm that the solution for the optimal SSKF goes to the optimal marginal solution. Recall that we solve for F and G by $F = \Sigma_{SS}A^T(A\Sigma_{SS}A^T + C)^{-1}$ and $G = P - FAP$ respectively, where Σ_{SS} is the fixed point solution to the Riccati equation, $\Sigma_{\mathrm{SS}} = P(\Sigma_{\mathrm{SS}} - \Sigma_{\mathrm{SS}}A^T(A\Sigma_{\mathrm{SS}}A^T + C)^{-1}A\Sigma_{\mathrm{SS}})P^T + Q$.

As $P \to 0$, most of the Riccati equation is eliminated, leaving only the Q term, so $\Sigma_{SS} \to Q$. This remaining covariance term is precisely the marginal covariance Σ_x when $P \to 0$ (by equation $\Sigma_x = P\Sigma_x P^T + Q$). The solution for F then becomes $F = \Sigma_x A^T(A\Sigma_x A^T + C)^{-1}$, which is the same as the solution for F obtained by solving for the marginal solution for F as done in appendix A. And trivially, the solution for $G \to 0$ as $P \to 0$. We see that the optimal decoder without prior dependencies becomes the optimal marginal decoder.

Furthermore, we can simulate a channel ($x \to y \to \hat{x}$) with x drawn from an AR(1) process and run the algorithm to optimize the encoder-decoder pair for a fixed value of the penalty parameter, λ. We can

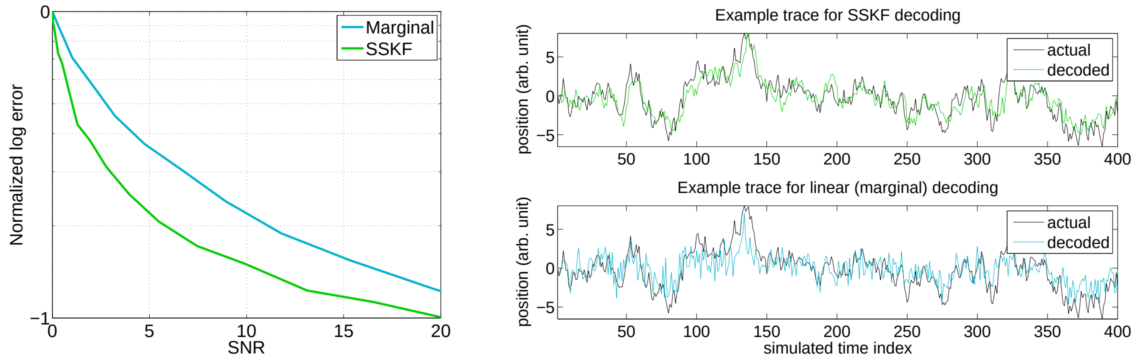

(a) (b)

Fig. S2: **Demonstration of decoding performance of optimized encoder-decoder schemes. We compare performance using the linear i.i.d. decoder which is optimized for marginal statistics vs. the SSKF decoder which is optimized for AR(1) prior structure. (a) Curves correspond to the task error. The y-axis is log-scale with $10^0 = 1$ error reflecting the error obtained by using the mean value as the decoded estimate. As SNR increases, error decreases at different rates for the two decoding approaches. (b) Example traces of a kinematic variable show difference in decoding performance between methods when SNR $\approx$ 3 and $x \sim AR(1)$. Simulation here uses x_t to be dimension $n = 3$ (i.e. 3D kinematics, with only one dimension visualized in the traces) and y_t to be dimension $k = 5$ "electrode" channels. Overall, SSKF performs much better. Notice the SSKF decoder yields a visually far less noisy decoded estimate at the same SNR (this difference between traces can be expressed either as MSE or as a difference in of $r^2 = .7$ for SSKF vs. $r^2 = .51$ for marginal).**

perform this optimization and examine the simulated error of optimal encoder-decoder pairs as a function of various magnitudes of the λ for the $\mathcal{G}_{SNR}(A)$ penalty (see Fig S2).

We expect that properly treating the AR(1) prior should improve recovery, and indeed we see this improvement reflected in Fig S2 for moderate SNR levels. In the very high SNR regime, the AR(1) prior is unnecessary and $G \to 0$ – the signal is high enough that we can trust the observations and the prior becomes less useful. In lower SNR regimes, we rely more on the AR(1) prior structure and less on the observations. However, as $SNR \to 0$, we can do no better than simply guessing the mean value of the process yielding a maximal value that the error can reach.

Additional References

Michail M Konstantinov, P Hr Petkov, and Nicolai D Christov. Perturbation analysis of the discrete riccati equation. *Kybernetika*, 29(1):18–29, 1993.

Peter Lancaster and Leiba Rodman. *Algebraic riccati equations*. Oxford University Press, 1995.

Kaare Brandt Petersen and Michael Syskind Pedersen. The matrix cookbook, 2012. URL `http://homepages.dcc.ufmg.br/~assuncao/EstatCC/aulas/matrixcookbook.pdf`.
