# Supplementary figures and images for: Encoder-Decoder Optimization for Brain-Computer Interfaces
Source: PLoS Comput Biol. 2015 Jun 1;11(6):e1004288. doi: 10.1371/journal.pcbi.1004288 (PMC4451011; doi:10.1371/journal.pcbi.1004288)

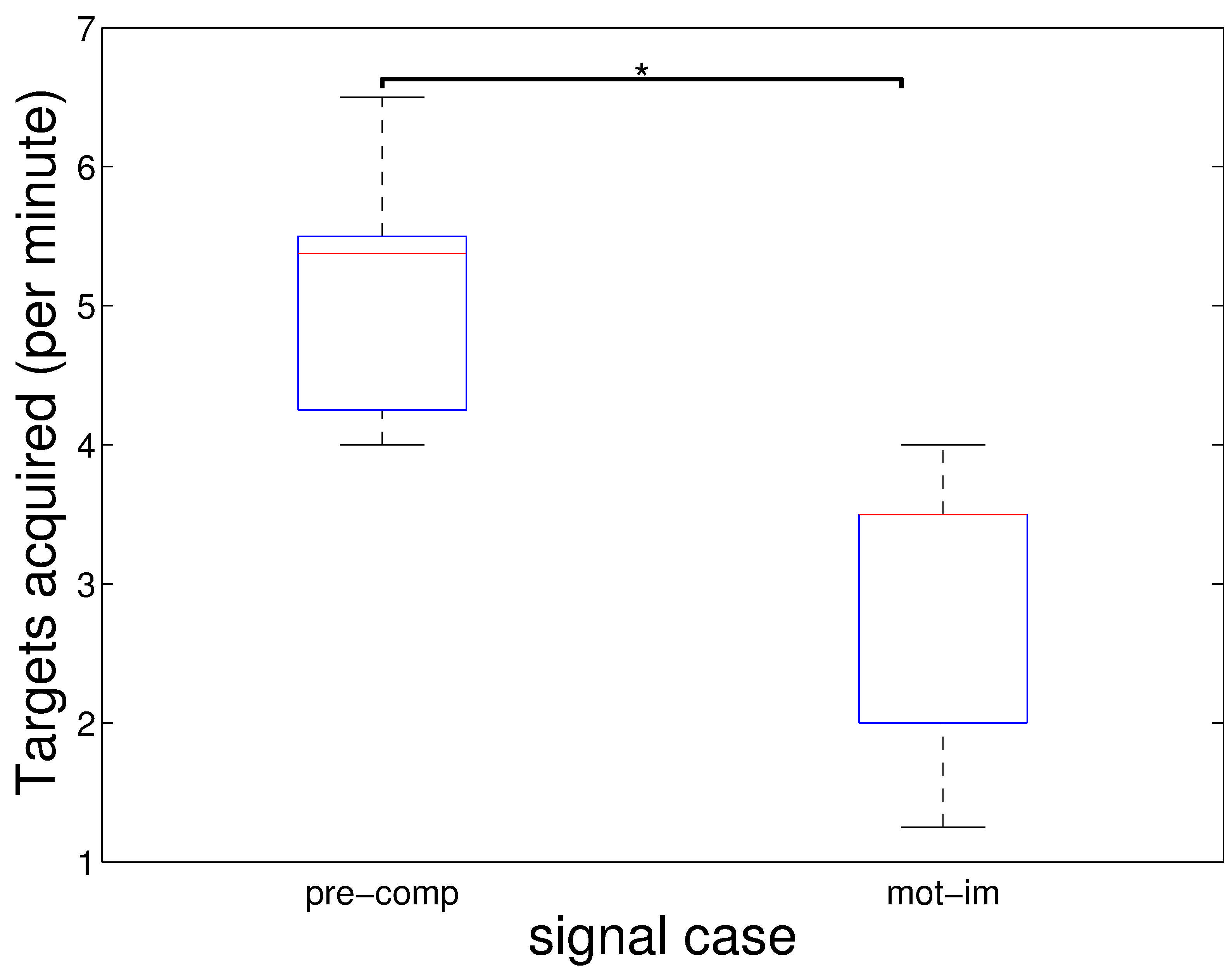

Supplement: S1 Fig — Two additional subjects were run for alternating blocks of testing with the motor-imitation and pre-computed decoder on signal case 1 (three tests blocks using each decoder per subject). The initialization for the motor-imitation decoder was run once at the beginning. Between decoder switches, subjects were given short exploration phases to refamiliarize themselves before testing. One subject began with the motor-imitation decoder, and the other began with the pre-computed decoder. Performance across subjects and blocks was significantly better using the pre-computed decoder (using a one-sided t-test on targets/time across repeats and subjects). Across subjects and repeats, no statistically significant linear trend was found for performance with the motor-imitation decoder. A weak trend was found for the pre-computed decoder across repeats (i.e. for these subjects there was some evidence that subjects continued to improve when using the pre-computed decoder p < .05) (TIFF) [file pcbi.1004288.s003.tiff]
